# Self-assembled dihydroartemisinin nanoparticles as a platform for cervical cancer chemotherapy

**DOI:** 10.1080/10717544.2020.1775725

**Published:** 2020-06-09

**Authors:** Yun Lu, Qian Wen, Jia Luo, Kang Xiong, ZhouXue Wu, BiQiong Wang, Yue Chen, Bo Yang, ShaoZhi Fu

**Affiliations:** a Department of Oncology, The Affiliated Hospital of Southwest Medical University, Luzhou, China; b Nuclear Medicine and Molecular Imaging Key Laboratory of Sichuan Province, Luzhou, China; c Department of Oncology, Three Gorges Central Hospital, Chongqing, China

**Keywords:** Dihydroartemisinin, MPEG-PCL, nanoparticles, cervical cancer

## Abstract

Dihydroartemisinin (DHA) is a potent anti-cancer drug that has limited clinical applications due to poor water solubility and low bioavailability. We designed a biodegradable poly(ethylene glycol) methyl ether-poly(ε-caprolactone) (MPEG-PCL) micelle carrier for DHA using the self-assembly method. The DHA/MPEG-PCL nanoparticles were spherical with an average particle size of 30.28 ± 0.27 nm, and released the drug in a sustained manner in aqueous solution. The drug-loaded nanoparticles showed dose-dependent toxicity in HeLa cells by inducing cycle arrest and apoptosis. Furthermore, compared to free DHA, the DHA/MPEG-PCL nanoparticles showed higher therapeutic efficacy and lower toxicity *in vivo*, and significantly inhibited tumor growth and prolonged the survival of tumor-bearing nude mice. In addition, the tumor tissues of the DHA/MPEG-PCL-treated mice showed a marked decline in the *in situ* expression of proliferation and angiogenesis markers. Taken together, the self-assembled DHA/MPEG-PCL nanoparticles are a highly promising delivery system for targeted cancer treatment.

## Introduction

Cancer is a leading cause of death in China, and the incidence and mortality of cervical cancer had increased significantly in recent years (Chen et al., 2016). It is a lethal gynecological tumor that is mainly treated by surgery, radiotherapy, and chemotherapy, of which the latter is used for patients with advanced or recurrent metastasis. With the development of more and more chemotherapeutic drugs and the improvement of treatment strategies, chemotherapy has achieved a certain effect on cervical cancer and improved the overall survival rate of locally advanced cervical cancer (Boardman et al., 2018). However, the main chemotherapeutic drugs have significant side effects (Wang et al., 2013), such as thrombocytopenia, hepatorenal toxicity, and neurotoxicity. Therefore, it is essential to explore new anti-cancer agents to improve the targeting of anti-tumor drugs and reduce the toxic side effects.

Plant-derived anti-cancer drugs have gained considerable attention in recent years with the identification of numerous botanical extracts and derivatives with anti-cancer properties. Dihydroartemisinin (DHA) is an artemisinin derivative isolated from the traditional Chinese herb Artemisia annua, and has a specific endoperoxide bridge structure. Due to its safety and efficacy, DHA has been used to treat malaria and fever since several decades (Sun et al., 2014). Recent studies have also reported an anti-proliferative effect of DHA on lung cancer, breast cancer, colon cancer, and cervical cancer cell lines, with minimal cytotoxicity in normal cells (Zhang et al., 2012; Wu et al., 2013; Lu et al., 2018; Liu et al., 2018a). The anti-cancer effects of DHA are mainly attributed to its inhibition of angiogenesis, induction of cell apoptosis and modulation of tumor-related genes (Dell'Eva et al., 2004; Liu et al., 2019; Wan et al., 2019). At the molecular level, the cleavage of the iron- or heme-mediated peroxide bridge and generation of reactive oxygen species (ROS) is the possible mechanism of DHA toxicity (Sun et al., 2014). However, the clinical potential of DHA is limited due to its poor water solubility, short half-life, and uncontrolled release (Kumar et al., 2019; Li et al., 2019). Therefore, we attempted to extend the anti-cancer effects of DHA by using a suitable delivery system to increase its solubility in aqueous solution.

In recent years, multiple vehicles based on liposomes, hydrogels, nanoparticles, etc., have been developed to increase the aqueous solubility of anti-cancer drugs in order to achieve targeted delivery and sustained release *in vivo* (Liu et al., 2016). Polymer nanoparticles-based drug delivery systems can not only improve the water solubility of hydrophobic small molecule chemotherapeutic drugs, but also accumulate passively in tumor sites through the enhanced permeability and retention (EPR) effect, thereby enhancing pharmacological effects, reducing adverse reactions, and improving drug tolerance of patients (Acharya & Sahoo, 2011; Liu et al., 2018b). Therefore, much of the recent focus on anti-cancer therapeutics has been on improving the bioavailability of potent drugs by encapsulating them in polymeric carriers to form polymer nanoparticles (Liu et al., 2018b). Poly(ethylene glycol) methyl ether-poly(ε-caprolactone) (MPEG-PCL) is an amphiphilic di-block copolymer with a hydrophobic core (PCL) that can encapsulate hydrophobic drugs, and a hydrophilic shell (PEG) that enhances stability and improves water solubility of the enclosed drugs. In addition, MPEG-PCL is biodegradable, biocompatible, and releases the drugs in a sustained manner, making it a suitable nanocarrier (Gong et al., 2012; Yu et al., 2017; Wu et al., 2018).

We prepared DHA/MPEG-PCL nanoparticles by the self-assembly method. Compared with other methods for preparing drug-loaded polymer nanoparticles, such as solvent diffusion method, emulsion solvent evaporation method (Farokhzad et al., 2004; Zhang et al., 2010), etc., the preparation procedure used in this study was simple, without the use of any surfactants, mild synthesis conditions, good repeatability, low cost, and easy to expand production.

In this study, we investigated the *in vitro* and *in vivo* inhibitory effects of DHA/MPEG-PCL nanoparticles on cervical cancer. The related mechanisms were also explored.

## Materials and methods

### Reagents

ε-Caprolactone (ε-CL, Alfa Aesar, Reston, VA), poly(ethylene glycol) methyl ether (MPEG, Mn = 2000), stannous octoate [Sn(Oct)_2_], coumarin-6 (C6), and 3-(4,5-dimethyl-2-thiazolyl)-2,5-diphenyl-2H-tetrazolium bromide (methyl thiazolyltetrazolium, MTT) were purchased from Sigma-Aldrich (St. Louis, MO). Dimethyl sulfoxide (DMSO), methanol, and acetonitrile (HPLC grade) were supplied by Kelong Co. Ltd. (Chengdu, China). Dihydroartemisinin was purchased from Meilun Biotechnology Co. Ltd. (Dalian, China).

### Synthesis of MPEG-PCL copolymers

MPEG-PCL di-block copolymer (MPEG_2000_–PCL_2000_, Mn = 4000) was synthesized by ring-opening polymerization of ε-CL and MPEG (weight ratio = 1:1), using Sn(Oct)_2_ (account for 0.5% of total feed stock) as the catalyst (Shi et al., 2012; Wang et al., 2019b). Briefly, a predetermined amount of ε-CL and MPEG were transferred into a dry glass flask under nitrogen atmosphere, and a small amount of Sn(Oct)_2_ was added with mild agitation at 130 °C for 6 h. At the end of polymerization, the mixture was cooled to room temperature (RT) and dissolved in dichloromethane, and re-precipitated using AR grade excess cold petroleum ether. The resulting MPEG–PCL copolymer was vacuum-dried to a constant weight and stored in desiccators till further use.

### Preparation of DHA/MPEG-PCL

The self-assembled DHA/MPEG-PCL nanoparticles were prepared by solid dispersion method as previously described (Wang et al., 2019b), with 5%, 10%, and 15% free DHA, respectively. Briefly, a specified amount of MPEG-PCL copolymer and free DHA were completely dissolved in a round-bottomed flask with dichloromethane. The solution was then evaporated in a rotator evaporator at 37 °C in a water bath. The film formed on the flask walls was re-dissolved in pre-heated ultrapure water (60 °C) and filtered through a 0.22 μm filter to obtain a clear light-blue opalescent solution. Finally, the filtrate was freeze-dried to a white powder.

### Drug-loading and encapsulation efficiency of DHA/MPEG-PCL

Drug loading (DL) and encapsulation efficiency (EE) were determined by high-performance liquid chromatography (HPLC, Agilent1260, Agilent Technologies, Palo Alto, CA) using a reverse phase C18 column (4.6 × 150 mm, 5 μm) at 30 °C. The mobile phase consisted of acetonitrile and water at the ratio of 60:40 (v/v) with a flow rate of 1 ml/min. The detection wavelength was set to 216 nm. DL and EE were calculated by the following equations:

(1) $$\mathrm{DL}=\frac{\mathrm{drug}}{\mathrm{polymer}+\mathrm{drug}}\times 100\%$$

(2) $$\mathrm{EE}=\frac{\text{actual drug loading}}{\text{theoretical drug loading }}\times 100\%$$

### Physicochemical characterization of DHA/MPEG-PCL nanoparticles

The average particle size and zeta potential were analyzed by dynamic light scattering (DLS, NanoBrook 90Plus Zeta, Brookhaven, Holtsville, NY) at 25 °C. Each sample was measured three times and all data were showed in mean ± standard deviation (SD). The surface morphology of DHA/MPEG-PCL nanoparticles was observed by transmission electron microscope (TEM, Tecnai G2 F20, FEI, ‎Hillsboro, OR). The DHA/MPEG-PCL samples diluted in distilled water were placed on a nitrocellulose-coated copper grid, negatively stained using phosphotungstic acid and dried at RT before observing. The crystallinity of DHA, MPEG-PCL, and DHA/MPEG-PCL powders was analyzed by an X-ray diffractometer (XRD, BRUKER D8, Karlsruhe, Germany), respectively. The measurements were performed using graphite monochromatized Cu-Kα radiation (*λ* = 0.1542 nm, 40 kV, 35 mA). The samples were measured in the 2*θ* range from 10° to 70° at a scanning rate of 4°/min.

### Stability study of DHA/MPEG-PCL nanoparticles

The stability of DHA/MPEG-PCL nanoparticles was investigated by measuring particle size at different pH values and media. Freeze-dried DHA/MPEG-PCL nanoparticles were re-dissolved in phosphate buffer saline (PBS, pH = 7.4) with or without 10% serum, incubated at 4 °C, RT, and 37 °C for 2 h, respectively. The particle size was determined by DLS. Meanwhile, in order to simulate the acidic tumor microcirculation, the particle size of DHA/MPEG-PCL nanoparticles was also tested using PBS at a pH of 5.5.

### *In vitro* drug release behavior

The release of DHA from free DHA and DHA/MPEG-PCL nanoparticles was investigated by dialysis method *in vitro*. Briefly, 4 ml (2.5 mg/ml) free DHA dissolved in methanol, while freeze-dried DHA/MPEG-PCL nanoparticles with 10% DL (equivalent to 10 mg free DHA) re-dissolved in 4 ml PBS (pH = 7.4), and each solution was poured into separate dialysis bags (molecular weight cutoff, 8–14 kDa). The bags were incubated in 40 ml PBS (pH = 7.4) containing Tween 80 (0.5%, w/w) at 37 °C ± 0.5 °C with mild shaking (100 rpm). Two milliliters of the medium was aspirated after 0.5, 2, 4, 6, 8, 12, 24, 48, 72, 96, 120, 144, and 168 hours, and replaced with equal volume of pre-heated PBS (pH = 7.4). The aliquots were stored at –20 °C for HPLC analysis. All samples were analyzed at least three times.

### *In vitro* hemolysis

The *in vitro* hemolytic activity of DHA/MPEG-PCL nanoparticles was analyzed as previously described (Zheng et al., 2010). Briefly, 3–5 ml fresh rabbit whole blood was stirred in a small beaker with a glass stick to remove fibrin and poured into a centrifuge tube. The red blood cells (RBCs) were washed with normal saline (NS) until the supernatant was clear, and 0.2 ml aliquots of 2% erythrocyte suspension were dispensed into clean glass tubes. Five milliliters distilled water, NS, and different concentrations of nanoparticles (0.1 mg/ml, 1 mg/ml, and 5 mg/ml) were respectively added into different tubes and incubated at 37 °C. Three hours later, the erythrocyte suspension was centrifuged at 1500 rpm for 10 minutes, and hemolysis was determined based on the color of the supernatant.

### *In vitro* cellular uptake

Human cervical carcinoma (HeLa) cells were purchased from Beijing ZhongKe Quality Inspection Biotechnology Co. Ltd. (Beijing, China). HeLa cells were cultured in RPMI medium (Gibco, Grand Island, NY) supplemented with 10% heated-inactivated fetal bovine serum (FBS, Gibco, Grand Island, NY) and 1% penicillin–streptomycin at 37 °C in a 5% CO_2_/95% air humidified atmosphere. To track the cellular uptake of MPEG-PCL nanoparticles, the fluorescent probe C6 was encapsulated in MPEG-PCL by the solid dispersion method. HeLa cells were seeded into 24-well plates at the density of 1 × 10^4^ cell/well, and cultured for 12 hours. After incubating with blank medium, 500 μg/ml MPEG-PCL, 100 μg/ml free C6, and C6/MPEG-PCL (equivalent to 100 μg/ml free C6) for three hours, the cells were washed thrice with PBS (0.01 M, pH = 7.4) and observed under a fluorescence microscope (Olympus IX73, Tokyo, Japan).

### *In vitro* cytotoxicity assay

To evaluate drug-induced cytotoxicity *in vitro*, the cells were seeded in 96-well plates at the density of 5 × 10^4^ cell/well and incubated for 12 hours. The medium was replaced with fresh culture medium containing free DHA, DHA/MPEG-PCL particles (corresponding to 50, 25, 12.5, 6.25, and 3.125 μg/ml DHA), and MPEG-PCL (800, 400, 200, 100, 50, and 25 μg/ml), and the cells were cultured for another 48 hours. At predetermined time points, 20 μl MTT solution (5 mg/ml) was added to each well and the supernatants were removed four hours later. The formazan crystals were dissolved with 150 μl DMSO for 10 minutes, and the absorbance (*A*) of each well was measured at 490 nm using iMark microplate absorbance reader (Bio-Rad, Hercules, CA). Each sample was measured three times and cell viability was calculated by the following equation:

$$\text{Cell viability }(\%)=\frac{A_{\mathrm{treated}}-A_{\mathrm{zero}\;\mathrm{set}}}{A_{\mathrm{control}}-\;A_{\mathrm{zero}\;\mathrm{set}}}\times 100\%.$$

### Apoptosis and cell cycle analysis

HeLa cells were cultured to the logarithmic growth stage, and the medium was separately replaced with fresh RPMI containing 25 μg/ml free DHA (approximately IC50), DHA/MPEG-PCL (equivalent to 25 μg/ml free DHA), and blank MPEG-PCL. The cells (including those floating in the medium) were harvested 48 hours later, and split into two aliquots. One portion was stained using 300 μl binding buffer, 5 μl Annexin V-FITC, and 5 μl PI for 15 min in the dark at RT, and the apoptotic cells were analyzed by Epics XL flow cytometer (Beckman Coulter, Miami, FL). The second aliquot was fixed overnight with 75% alcohol at 4 °C, washed thrice with PBS, and stained with 500 μl PI/RNase Staining Buffer (BD Biosciences, San Diego, CA) for 15 min in the dark at RT. The cell cycle distribution was analyzed by flow cytometry. All tests were repeated thrice. The data were quantitatively analyzed by ModFit LT 3.1 software (Verity Software, Topsham, ME).

### *In vivo* antitumor effect

Female athymic BALB/C nude mice (4–5 weeks old, weighing 14–18 g) were purchased from TengXin Biotechnology Co. Ltd. (Chongqing, China), and housed in a temperature-controlled environment (20–22 °C) at relative humidity of 50–60% and 12 h light–dark cycles, with ad libitum access to standard laboratory chow and tap water. Nude mice were cared for in accordance with the Laboratory Animals Care and Use Guidelines of the National Institute of Health (NIH). All animal experiments were approved by the Institutional Animal Care and Treatment Committee of Southwest Medical University (Luzhou, China). Tumor xenograft model was established by subcutaneously injecting each mouse with 100 μl of HeLa cells (1.0 × 10^7^/ml) into their right thigh. Once the tumors grew to ∼100 mm^3^, the tumor-bearing mice were randomly divided into the control, MPEG-PCL, free DHA, and DHA/MPEG-PCL groups (*n* = 10 each), and respectively injected with NS, MPEG-PCL, 20 mg/kg free DHA, and DHA/MPEG-PCL (equivalent to 20 mg/kg free DHA) through the tail vein every two days for a total of five times. The tumor size and weight were monitored every other day from the start of treatment, and the tumor volume was calculated as $\frac{1}{2}$×length × width^2^. Three mice from each group were sacrificed by cervical dislocation on day 10, the tumors and major organs were harvested. The remaining mice were observed for survival and tumor progression.

### Micro ^18^F-FDG PET/CT imaging

Glucose metabolism in the tumor tissues was determined using micro ^18^F-FDG PET/CT imaging scans (Siemens, Munich, Germany). At the end of the treatment regimen, the tumor-bearing mice were fasted for at least eight hours and anesthetized by an intraperitoneal injection of 1% pentobarbital (5 ml/kg). Each mouse was then injected with 100–200 μCi FDG through the tail vein, and placed in the center of the PET/CT imaging field 30 minutes later. Images were acquired with 10 min per bed position. According to a previous report (You et al., 2016), the image plane with the largest tumor appearance on the PET/CT fusion image was chosen for data collection. The irregular region of interest (ROI) covering the entire tumor was manually plotted to obtain the standardized uptake value (SUV).

### Immunohistochemistry and H&E staining

The tumors and major organs were fixed in 10% neutral buffered formalin solution for 24 hours, embedded in paraffin and cut into 3–4 μm thick sections. The heart, liver, spleen, lung, and kidney tissue sections were stained with hematoxylin and eosin (H&E) and observed by two pathologists in a blinded manner. The tumor tissue sections were stained with anti-human Ki-67, CD31, and VEGF antibodies as per standard protocols, and the slides were examined under a microscope (Olympus IX73, Tokyo, Japan) at ×400 and ×200 magnification. The proportion of Ki67+ and VEGF + cells relative to the total number of cells, and the number of CD31+ micro-vessels were counted in five randomly selected fields per sample, and the mean values were calculated.

### Statistical analysis

Statistical analysis was performed using GraphPad prism version 6.0 software (GraphPad Software, San Diego, CA). Student’s *t*-test and one-way ANOVA were respectively used to compare two or multiple groups. *p*<.05 was considered statistically significant.

## Results

### Preparation and characterization of DHA/MPEG-PCL nanoparticles

**Figure 1.**
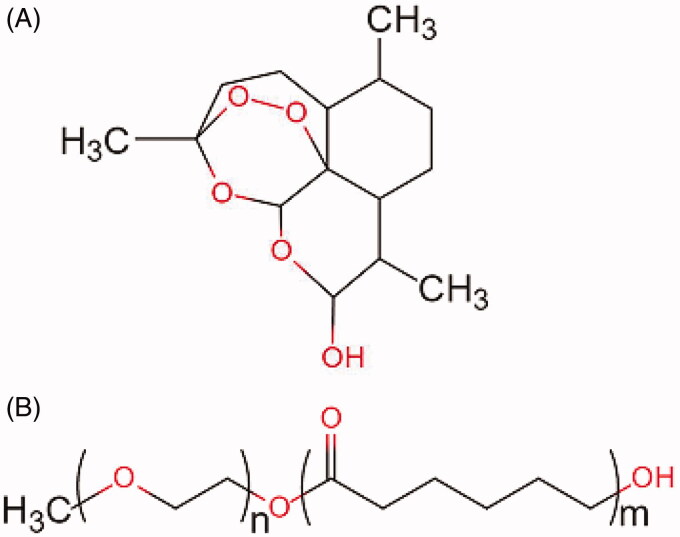
Molecular structures of DHA (A) and MPEG-PCL copolymer (B).

**Figure 2.**
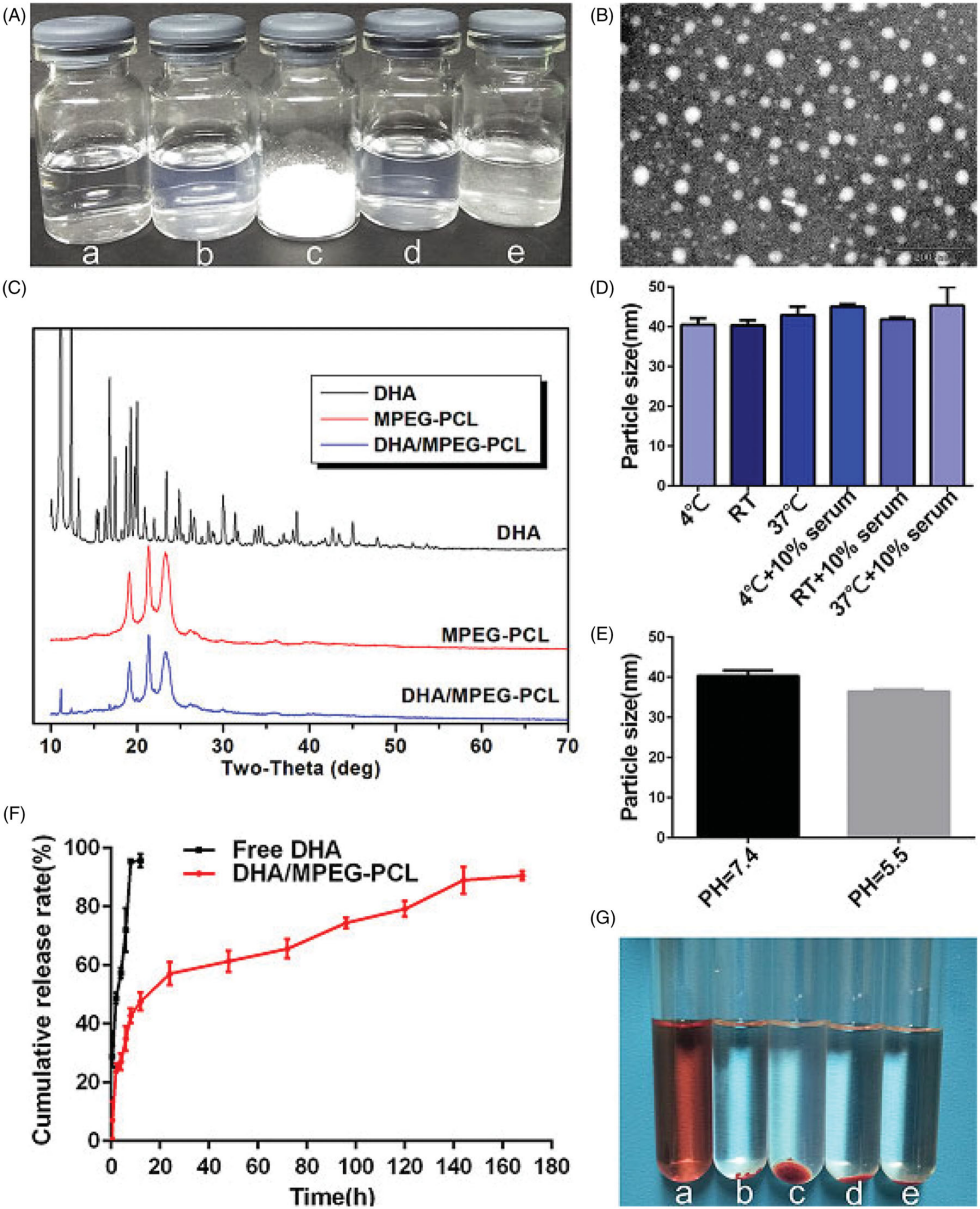
(A) Compare the appearance of several solutions (a: NS, b: DHA/MPEG-PCL nanoparticles, c: freeze-dried powder of DHA/MPEG-PCL nanoparticles, d: re-dissolved DHA/MPEG-PCL nanoparticles, e: free DHA in water). (B) TEM image of DHA/MPEG-PCL nanoparticles. (C) XRD analysis of DHA, MPEG-PCL micelles, and DHA/MPEG-PCL nanoparticles. (D) Particle size of the DHA/MPEG-PCL nanoparticles under different conditions. (E) Particle size of the DHA/MPEG-PCL nanoparticles at different pH values. (F) *In vitro* drug release of free DHA and DHA/MPEG-PCL nanoparticles. (G) Hemolytic test on DHA/MPEG-PCL nanoparticles (a: distilled water, b: NS, c–e: the DHA/MPEG-PCL nanoparticles with concentrations of 5 mg/ml, 1 mg/ml, and 0.1 mg/ml, respectively).

The molecular structures of DHA and MPEG-PCL copolymer are shown in Figure 1. The DHA/MPEG-PCL nanoparticles with different DHA contents are listed in Table 1. The particle sizes of all samples were smaller than 40 nm with good polydispersity index (PDI). The properties of MPEG-PCL nanoparticles were not significantly changed after DL. Due to the loss in the preparation process and the difference in physicochemical properties (such as molecular weight, crystallinity, etc.) between DHA and MPEG-PCL, the drug cannot be completely encapsulated into nanoparticles. The 10% DHA-encapsulated nanoparticles showed optimum EE (74.9 ± 0.56%) compared to the particles containing 5% (72.9 ± 5.94%) and 15% (68.87 ± 1.09%) DHA. Therefore, the 10% DHA/MPEG-PCL nanoparticles were selected for further experiments. As shown in Figure 2(A), the nanoparticle solution had a blue opalescence (b) and could be freeze-dried into a white powder (c), which re-dissolved stably into an evenly dispersed solution with a blue opalescence (d). However, the free drug was not completely dissolved in water and formed a turbid suspension (e). TEM images also showed a uniform spherical shape of the nanoparticles (Figure 2(B)). The XRD spectra are shown in Figure 2(C). It could be found that the characteristic X-ray diffraction peaks of DHA almost disappeared in the spectrum of DHA/MPEG-PCL, except that there was a peak with significantly reduced diffraction intensity around 11°. It indicated that DHA lost its original crystal structure and mainly existed in an amorphous state in the DHA/MPEG-PCL nanoparticles.

**Table 1.** Characteristics of DHA/MPEG-PCL.

Samples	Theoretical DL (%)	DL (%)	EE (%)	Size (nm)	PDI	Zeta potential (mV)
1	0	0	0	38.6 ± 1.36	0.23 ± 0.001	–3.56 ± 0.29
2	5%	3.65 ± 0.29	72.9 ± 5.94	35.77 ± 0.27	0.2 ± 0.01	–1.96 ± 1.55
3	10%	7.49 ± 0.06	74.9 ± 0.56**	30.28 ± 0.27	0.12 ± 0.02	–4.37 ± 2.77
4	15%	10.33 ± 0.17	68.87 ± 1.09	36.49 ± 1.29	0.17 ± 0.01	–6.69 ± 9.15

** *p*<.01 compared with the sample 4.

### *In vitro* stability of DHA/MPEG-PCL nanoparticles

As shown in Figure 2(D), after 2 h of treatment under various conditions, the difference in particle size of DHA/MPEG-PCL nanoparticles was not statistically significant, indicating that DHA/MPEG-PCL nanoparticles were stable under daily storage, serum addition, and physiological conditions, which could meet the requirement of clinical application. Furthermore, under the same conditions, the particle size of DHA/MPEG-PCL nanoparticles did not change significantly at pH of 7.4 and 5.5 (Figure 2(E)), indicating that pH value of media would not affect the stability of the nanoparticles.

### Blood compatibility and release manner of DHA/MPEG-PCL nanoparticles

The blood compatibility of DHA/MPEG-PCL nanoparticles was evaluated in terms of hemolysis. As shown in Figure 2(G), the RBCs incubated with distilled water were completely lysed (a), whereas the supernatants were absolutely achromatic in the presence of NS (b) and various concentrations of the nanoparticles (c, d, and e), indicating no significant hemolysis phenomenon. In addition, the release behavior of DHA is shown in Figure 2(F), free DHA was nearly completely released within 12 h, whereas 90.53 ± 1.55% of the DHA was released from DHA/MPEG-PCL nanoparticles over a period of seven days. By comparing the cumulative release rate, it could be seen that DHA/MPEG-PCL exhibited a controlled and sustained release behavior. Taken together, the DHA/MPEG-PCL nanoparticles are non-hemolytic, and can release DHA in a sustained manner.

### MPEG-PCL nanoparticles increased cellular uptake and enhanced DHA cytotoxicity *in vitro*

**Figure 3.**
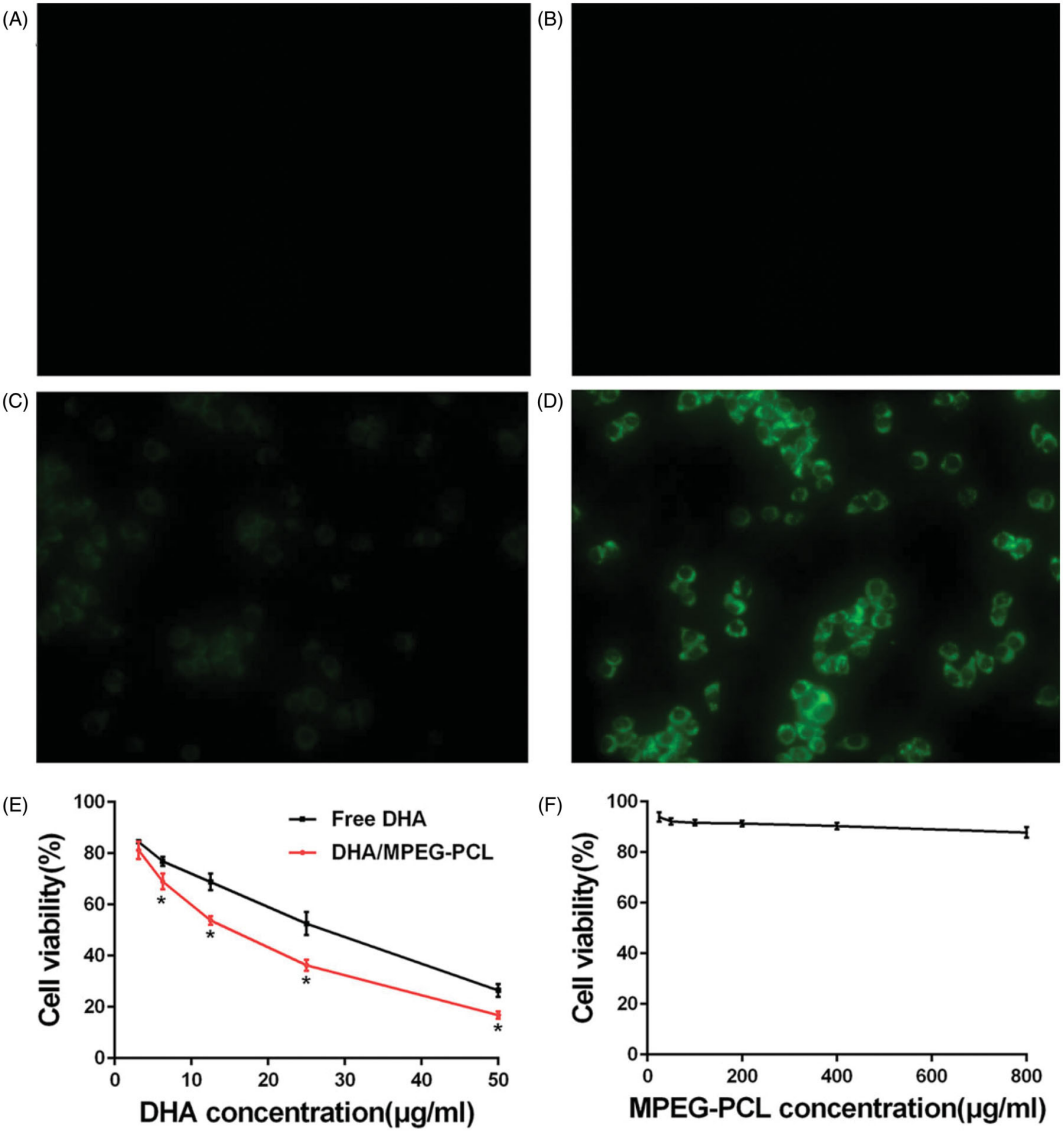
*In vitro* analysis of cytotoxicity and cell uptake. Fluorescent images of HeLa cells treated with (A) medium, (B) MPEG-PCL micelles, (C) free C6, and (D) C6/MPEG-PCL nanoparticles for 3 h (×400). (E) Cell viability of HeLa cells treated with free DHA and DHA/MPEG-PCL nanoparticles at the same DHA dose for 48 h. *p<.05. (F) Cell viability of HeLa cells cultured with different concentrations of the MPEG-PCL micelles for 48 h.

HeLa cells incubated with the C6/MPEG-PCL particles showed significantly higher fluorescence intensity (Figure 3(D)) compared to those treated with free C6 (Figure 3(C)), whereas the control (Figure 3(A)) and blank carrier (Figure 3(B)) groups showed no fluorescence. Furthermore, the blank MPEG-PCL did not exhibit any significant cytotoxicity, since more than 85% of the HeLa cells survived when incubated with 800 µg/ml of the carrier (Figure 3(F)), indicating that MPEG-PCL carrier was relatively nontoxic to HeLa cells. In contrast, both free DHA and DHA/MPEG-PCL decreased the viability of HeLa cells in a concentration-dependent manner, with the encapsulated form showing a greater cytotoxic effect compared to the free drug, especially when the concentration of DHA was at least 6.25 μg/ml (*p*<.05; Figure 3(E)). Taken together, the MPEG-PCL nanoparticles enhanced cellular uptake of the cargo and significantly improved the inhibitory effect of DHA on HeLa cells.

### *In vitro* cell apoptosis and cell cycle analysis

**Figure 4.**
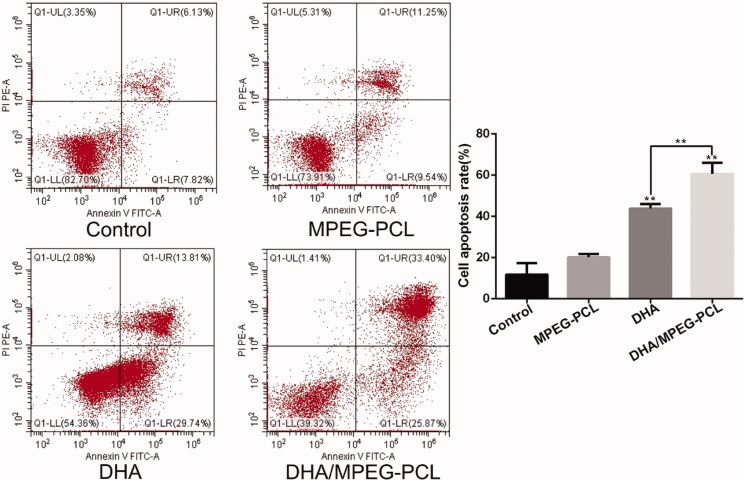
Flow cytometry used to detect the apoptosis of HeLa cells with different treatments, and the percentage of apoptosis in each group. The concentration of DHA was 25 μg/ml and the processing time was 48 hours. **p<.01.

**Figure 5.**
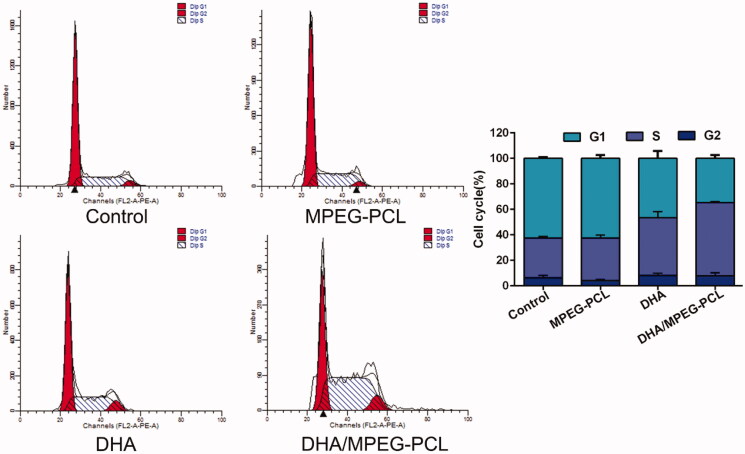
Flow cytometry used to detect the cell cycle of HeLa cells with different treatments, and the percentage of each phase in each group. The concentration of DHA was 25 μg/ml and the processing time was 48 hours.

The mechanism of DHA inhibited the proliferation of HeLa cells which was investigated by apoptosis and cycle analysis. Free DHA and DHA/MPEG-PCL significantly increased the percentage of apoptotic HeLa cells to 43.68 ± 2.3% and 60.51 ± 5.39% respectively compared to the untreated controls (11.73 ± 5.58%, *p*<.01) and the blank carrier group (20.13 ± 1.55%, *p*<.01). In addition, the apoptosis rate was significantly higher in the DHA/MPEG-PCL versus free DHA-treated cells (*p*<.01) and no significant difference between the control group and the carrier group (*p*>.05). These could be intuitively seen from Figure 4. The difference in cell cycle between each group is shown in Figure 5, compared to the control (31.27 ± 0.88%, *p*<.05) and the carrier (33.39 ± 2.57%, *p*<.05) group, the proportion of cells in the S-phase increased significantly when treated with free DHA (45.32 ± 4.9%) or DHA/MPEG-PCL (57.51 ± 0.74%). Combined with the results of apoptosis, it is speculated that a G1/S arrest might have occurred. The results revealed that DHA inhibited the proliferation of HeLa cells by inducing apoptosis and cycle arrest. The DHA/MPEG-PCL nanoparticles had a stronger induction effect than free DHA.

### DHA/MPEG-PCL anti-tumor effect and toxicity evaluation *in vivo*

**Figure 6.**
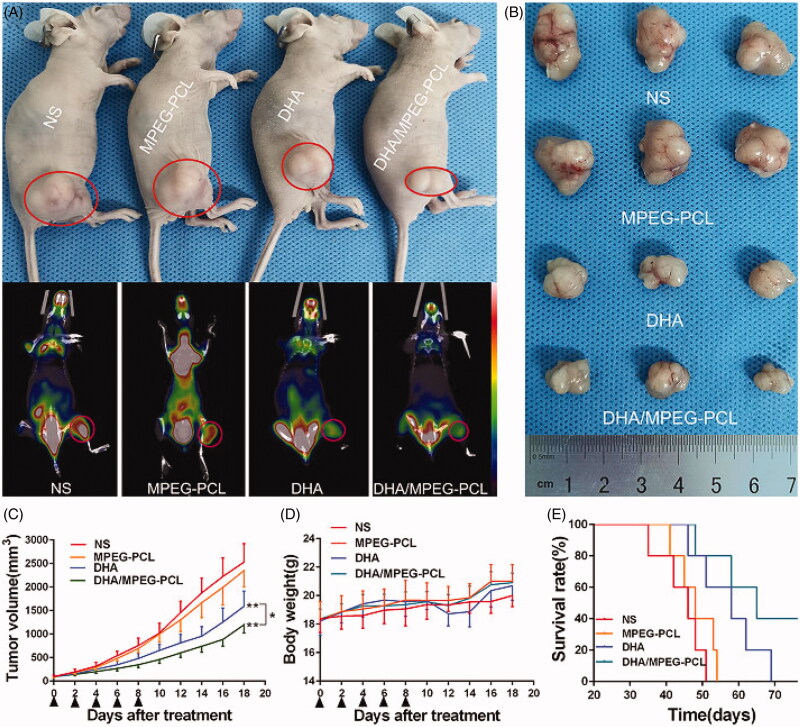
*In vivo* anti-cancer effect of free DHA and the DHA/MPEG-PCL nanoparticles. (A) General view of representative tumor-bearing mice in each group and corresponding ^18^F-FDG PET/CT images. The red circles represent the tumor location. (B) Excised tumors from mice in each group at day 10 after treatment (*n* = 3). (C) Tumor growth curves and (D) body weight changes of mice in each group during the experiment. The black triangles represent the time points at which treatment were received. *p<.05; **p<.01. (E) Survival curves in each group.

**Figure 7.**
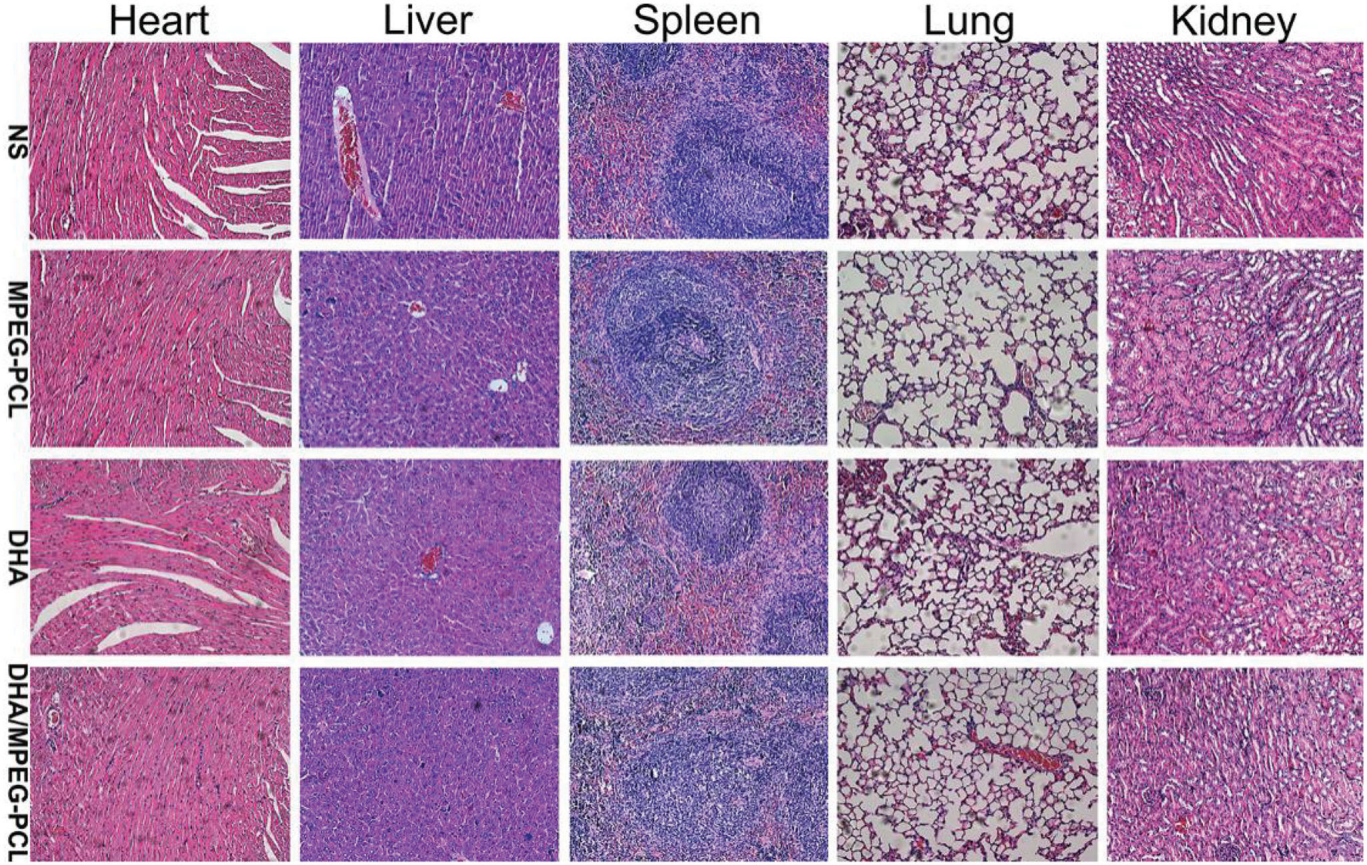
Tissue toxicity assessment. Representative images of H&E staining of heart, liver, spleen, lung, and kidney in each group (×200).

The *in vivo* efficacy of DHA/MPEG-PCL was evaluated in terms of tumor volume, metabolism, and the survival of the tumor-bearing mice. The mean maximum standardized uptake value (SUVmax) of glucose was significantly lower in the free DHA (1.53 ± 0.15) and DHA/MPEG-PCL (1.2 ± 0.1) treated mice compared to the NS (2.17 ± 0.12) and MPEG-PCL (2.07 ± 0.06) (*p*<.01) groups. In addition, the encapsulated DHA resulted in significantly lower ^18^F-FDG uptake compared to the free drug (*p*<.05; Figure 6(A)). As shown in Figure 6(B,C), the tumor growth was significantly slower in the mice treated with free DHA group or DHA/MPEG-PCL compared to the NS and MPEG-PCL groups (*p*<.01), and the inhibitory effect of DHA/MPEG-PCL was stronger compared to free DHA (*p*<.05). Furthermore, as shown in Figure 6(E), compared with the control group, the median survival time of mice in the DHA group (58 days, *p*<.05) and the DHA/MPEG-PCL group (65 days, *p*<.01) was statistically significant. There was no statistical significance between the control group (46 days) and the carrier group (48 days). Finally, the body weight of mice did not show any notable differences across the groups (Figure 6(D)), and no significant pathological changes or inflammatory lesions were seen in the major organs (Figure 7). Taken together, the DHA/MPEG-PCL nanoparticles showed significant *in vivo* therapeutic effect with minimal systemic toxicity.

**Figure 8.**
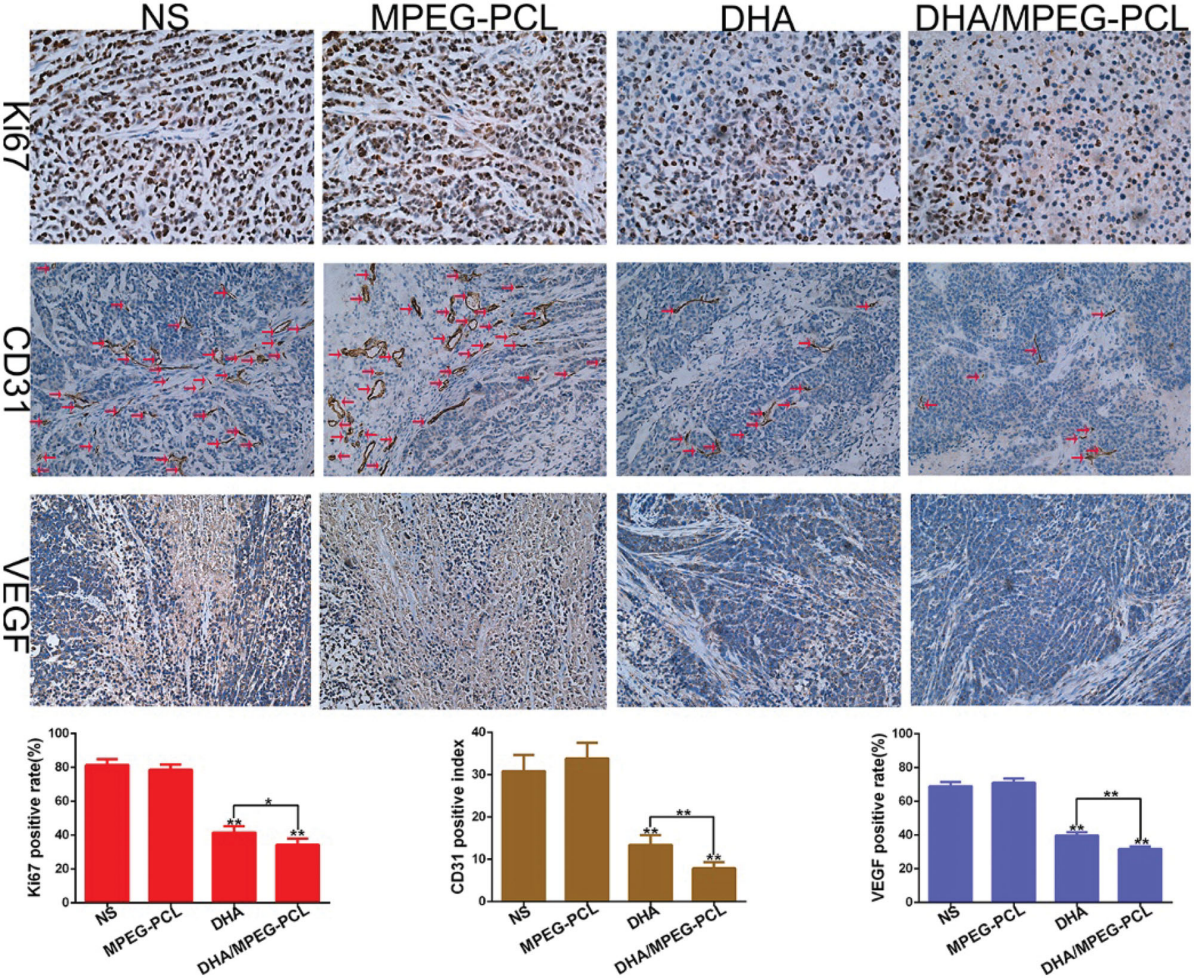
Representative images of immunohistochemical analysis of each group for the evaluation of Ki67 (×400), CD31 (×200), and VEGF (×200), and quantitative analysis of corresponding items. The red arrows represent the location of blood vessel. *p<.05; **p<.01.

As shown in Figure 8, the percentage of Ki67+ proliferative cells in the tumors of DHA/MPEG-PCL mice (34.21 ± 3.73%) was significantly lower compared to that in the DHA group (41.48 ± 3.85%, *p*<.05), and increased markedly in the NS group (81.27 ± 3.44%, *p*<.01) and the MPEG-PCL group (78.56 ± 3.02%, *p*<.01). CD31 immunostaining was used to assess tumor angiogenesis, and the vascular density in the DHA/MPEG-PCL (7.8 ± 1.48) and DHA (13.4 ± 2.3) groups was significantly lower than that in the NS group (30.8 ± 3.83, *p*<.01) and the MPEG-PCL group (33.8 ± 3.7, *p*<.01). Consistent with this, VEGF protein expression levels were also significantly lower in the tumors of the DHA/MPEG-PCL group (31.6 ± 1.54) compared to the other groups (*p*<.01). It was suggested that DHA played the role of anti-cervical cancer *in vivo* by inhibiting angiogenesis and tumor cell proliferation, and the inhibition effect of DHA/MPEG-PCL nanoparticles was better.

## Discussion

DHA is an active metabolite of artemisinin derivatives, and FDA approved for treating malaria (Wang et al., 2019a). Its potential as a therapeutic agent against cancer has also been explored in recent years (Liu et al., 2015). It inhibits proliferation and angiogenesis in tumors, and induces apoptosis and cycle arrest in various cancer cell lines (Ma et al., 2015). Apoptosis may be related to DHA-induced nuclear activation of P53 and the down-regulation of NQO1, activating the mitochondrial-related cell apoptosis pathway (Zhang et al., 2017). In addition, one study suggested that DHA may cause cell cycle arrest by suppressing FOXM1 (Lin et al., 2016). However, its clinical applications are limited due to its poor solubility and low bioavailability (Duan et al., 2019). Therefore, the development of an intravenously injectable aqueous formulation of DHA is of great significance.

Previously, some studies have been conducted on formulations to improve DHA performance. Among them, gelatin or hyaluronan nanoparticles had a very low EE (13% or 35%) (Sun et al., 2014); although the formulation of lipid nanoparticles obtained a high EE (>90%), the preparation process required mechanical stirring and the final formulation had a large particle size (>100 nm) (Zhang et al., 2010; Wang et al., 2019c). The formulation of PLGA nanoparticles with phospholipid was novel and interesting, but the preparation process was relatively complicated, multiple organic solvents were used, and the particle size was also large (265.3 ± 7.9 nm) (Wang et al., 2016b). In contrast, the DHA/MPEG-PCL nanoparticles we prepared had relatively good EE (74.9 ± 0.56%), small particle size (30.28 ± 0.27 nm), high safety, and easy industrialization. In this study, we encapsulated DHA in MPEG-PCL micelles, which is a highly suitable nanoscale drug delivery system due to its simple synthesis process, safety, and biodegradability (Yu et al., 2017). DHA was encapsulated into the carrier by a simple self-assembly method. The bi-block MPEG-PCL polymer is a semi-crystalline polymer with low glass transition temperature. When temperature increased to near the glass transition temperature, it changed from semi-crystalline to amorphous state, and the activity capacity of polymer chains increased, which allowed amphiphilicity triggered MPEG-PCL micellization and encapsulated the hydrophobic drug (Gou et al., 2009). Owing to the amphiphilicity of MPEG-PCL copolymer, the hydrophobic PCL chains formed a ‘core’ to encapsulate insoluble DHA, and the hydrophilic PEG chains formed a ‘shell’ to provide excellent water dispersity (Gou et al., 2011; Qiu et al., 2013).

The self-assembled DHA/MPEG-PCL nanoparticles were of spherical shape with a narrow size distribution, and the lyophilized nanoparticles were highly stable and dissolved to form a uniformly dispersed solution suitable for intravenous injection. Previous studies show that nanoparticles smaller than 100 nm can evade renal filtration and circulate for a longer time in the blood to generate the EPR effect (Xu et al., 2015; Wang et al., 2019c). In addition, consistent with previous findings, DHA was initially discharged rapidly from the MPEG-PCL nanoparticles followed by a slower and sustained release (Zhang et al., 2010; Wang et al., 2016b). The initial burst is likely due to the diffusion of surface-bound drug from the nanoparticles to release medium, and the DHA situated in the PCL core is released thereafter due to the degradation or hydrolysis of nanoparticles (Danafar et al., 2014). Degradation first occurred at the interface between the MPEG shell and the PCL core, causing part of the PEG chains to detach. Then, as the degradation process progressed, random break in the PCL chains caused degradation inside the PCL core and DHA was released (Jiang et al., 2008). DHA showed dose-dependent toxicity in HeLa cells (Wang et al., 2016a; Zhang et al., 2017), which was significantly augmented following its encapsulation in MPEG-PCL since the micellar structure of the latter enhances cellular uptake. Thus, the DHA/MPEG-PCL nanoparticles had the unique properties of enhanced water solubility, enhanced uptake, sustained drug release, and passive targeting to tumors, all of which might potentially improve the bioavailability of DHA *in vivo* and enhance its anti-cervical cancer effect.

The *in vivo* anti-cancer effect of DHA/MPEG-PCL was evaluated in a HeLa xenograft model through ^18^F-FDG PET/CT imaging and survival analysis. ^18^F-FDG PET/CT is used to monitor early treatment response and prognosis (Zhang et al., 2018) in terms of tumor FDG uptake, which is indicative of glucose metabolism and an indirect measurement of tumor growth. The mice treated with DHA/MPEG-PCL showed the lowest tumor FDG uptake, which corresponded to significantly prolonged survival compared to the untreated as well as the free DHA-treated mice. This potent anti-tumor effect may be related to the small size and solubility of these nanoparticles, as well as the EPR effect. These features enhanced the absorption of nanoparticles at the tumor sites and thus allowed the drug to accumulate and release in a sustained fashion in the target tissues. The pro-apoptotic effect of DHA in HeLa cells has been reported previously (Zhu et al., 2014). Consistent with the findings so far, the DHA/MPEG-PCL nanoparticles resulted in higher apoptosis rates and S-phase arrest, as well as stronger anti-proliferative effects. The anti-angiogenic effects of DHA were also confirmed in our study with lower VEGF expression and fewer CD31-positive micro-vessels in the suitably treated mice.

The clinical application of DHA/MPEG-PCL nanoparticles is incumbent on their biosafety. The DHA/MPEG-PCL nanoparticles did not cause any hemolytic reaction or induce significant toxicity and inflammation in the healthy tissues. No changes were observed in the food/water intake or fur quality of the mice during the treatment regimen. In addition, previous study has shown that the MPEG-PCL polymer has less effect on the cell viability of both L929 and HEK293 cells at concentrations below 2 mg/ml, and that intravenous injection of 2 g/kg MPEG-PCL polymer does not cause acute toxic injury in rats (Gou et al., 2009). In our study, the dose we used *in vivo* is less than 2 g/kg. Meanwhile, no toxicity associated with the use of DHA was found in animals or humans. Therefore, the DHA/MPEG-PCL nanoparticles are a highly promising therapeutic agent against cervical cancer due to its excellent antitumor efficacy and high safety.

To summarize, the DHA/MPEG-PCL nanoparticles were stable, biocompatible, and easy to synthesize, and showed enhanced anti-tumor efficacy through passive targeting. Our subsequent efforts will focus on improving DL and designing active targeting systems with optimal specificity to cervical cancer cells or microenvironment, such as folate targeting, ROS response, etc. In addition, the detailed anti-tumor mechanisms of the DHA/MPEG-PCL nanoparticles including involved genes or proteins also need to be explored.

## Conclusions

We designed a passively targeted drug delivery system for DHA by self-assembling the biodegradable copolymer MPEG-PCL. The DHA/MPEG-PCL nanoparticles showed enhanced therapeutic efficacy by inducing apoptosis and cycle arrest, and inhibiting proliferation and angiogenesis, indicating their clinical potential.
