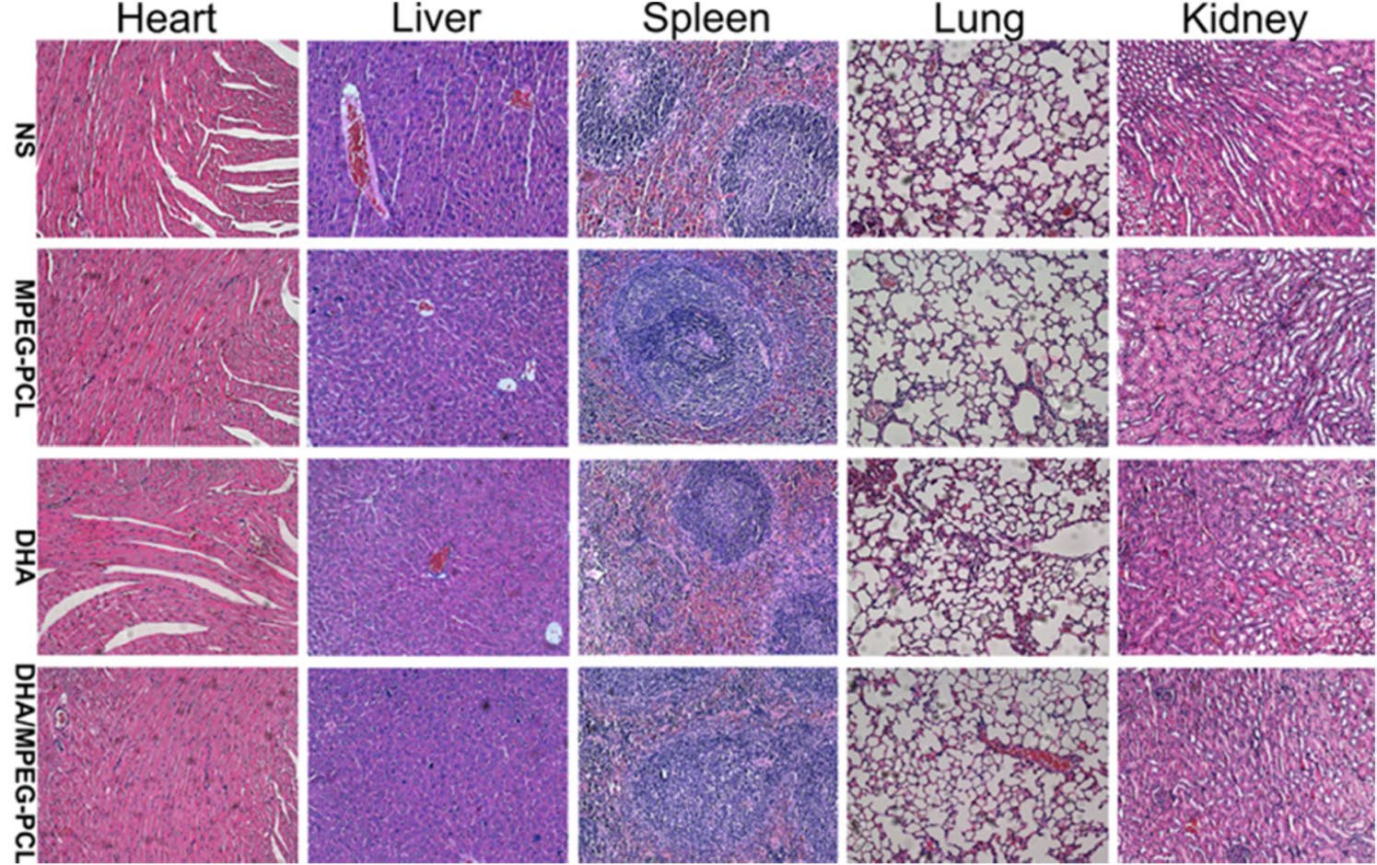# Correction

**DOI:** 10.1080/10717544.2024.2374134

**Published:** 2024-07-02

**Article title:** Article Self-assembled dihydroartemisinin nanoparticles as a platform for cervical cancer chemotherapy

**Authors:** Yun Lu, Qian Wen, Jia Luo, Kang Xiong, ZhouXue Wu, BiQiong Wang, Yue Chen, Bo Yang & ShaoZhi Fu

**Journal:** *Drug Delivery*

**Bibliometrics:** Volume 27, Number 01, pages 876-887

**DOI:** https://doi.org/10.1080/10717544.2020.1775725

When the above article published online, it contained incorrect Figure 7. This has been corrected and republished with the correct figure. The correct figure 7 is given below.